# Mechanisms and parameters of cryotherapy intervention for early postoperative swelling following total knee arthroplasty: A scoping review

**DOI:** 10.1002/jeo2.70197

**Published:** 2025-03-07

**Authors:** Lin Yang, Yi‐fang Zhan, Zan‐jing Zhai, Hong Ruan, Hui‐Wu Li

**Affiliations:** ^1^ Department of Nursing Shanghai Ninth People's Hospital, Shanghai JiaoTong University School of Medicine Shanghai China; ^2^ School of Nursing Shanghai JiaoTong University Shanghai China; ^3^ Department of Orthopaedic Shanghai Ninth People's Hospital, Shanghai JiaoTong University School of Medicine Shanghai China; ^4^ Shanghai Nursing Association Shanghai China

**Keywords:** cryotherapy, parameter, scoping review, swelling, total knee arthroplasty

## Abstract

**Purpose:**

Swelling after total knee replacement surgery can hinder recovery, cryotherapy is one of the non‐pharmacological interventions. However, the evidence of effectiveness is limited, possibly due to the heterogeneity of parameters. This scoping review aims to summarise existing evidence, clarify the mechanism and effect of cryotherapy on swelling after total knee arthroplasty, and analyze various parameters, providing evidence for clinical practice and future research.

**Methods:**

A literature search was performed on PubMed to include articles which reported on the cryotherapy impacts postoperative swelling after total knee arthroplasty. Snowballing research was used to obtain more sources.

**Results:**

A total of 69 studies were identified from the initial research, of which 40 articles were included for the full text analysis. Cryotherapy primarily acts on swelling by reducing haemorrhage and inflammatory responses. The level of evidence for the effectiveness of cryotherapy is low, and there is no standard in its parameters. The initiation of cryotherapy is increasingly recommended to start immediately after surgery. The selection of treatment temperature needs to balance efficacy and safety, but measuring intra‐articular temperature presents obstacles, making skin temperature a more feasible option. However, it is unclear how to achieve the desired skin temperature by setting a combination of treatment temperature, pressure and duration. When determining the length of the interval, particular attention should be paid to the changes in blood perfusion levels during the rewarming phase, as evidence suggests that skin temperature during rewarming may not accurately reflect the actual level of blood perfusion. The location and duration of cryotherapy can be preliminarily determined through existing evidence and mechanism analysis.

**Conclusion:**

Some cryotherapy parameters are supported by evidence and can be practiced in clinical practice. It should be noted that skin temperature has limitations as an observation indicator during the rewarming stage, and the frequency of cold therapy needs further research to determine.

**Level of Evidence:**

Level IV.

**Abbreviation**
TKA: total knee arthroplasty

## INTRODUCTION

Total knee arthroplasty (TKA) is one of the most effective surgical treatments for end‐stage osteoarthritis, and enhanced recovery a key strategy for improving the quality of orthopaedic care [19]. However, postoperative lower limb swelling is a significant challenge, becomes a common cause of patient dissatisfaction and pain, with 90.7% of patients experiencing swelling 2–3 weeks after discharge [29]. Swelling could induce joint‐derived muscle inhibition, further weakening quadriceps strength, hindering early rehabilitation exercises and increasing the risk of other complications [1, 33]. Therefore, postoperative swelling after TKA has become a shared concern among healthcare providers.

To address the issue of swelling after TKA, cryotherapy is commonly applied due to its proven effectiveness in preventing swelling following other acute surgical injuries [16]. While there is evidence that cryotherapy can reduce postoperative bleeding, its effectiveness in controlling swelling remains inconsistent. Some studies suggest cryotherapy reduces swelling, but conclusions are not universally agreed upon [32]. A key factor impeding the synthesis of evidence is the lack of consistency in the parameters (e.g. temperature, duration, and frequency) used in different studies [4, 20, 35]. This heterogeneity in cryotherapy settings complicates comparisons and the development of a standardised approach. Setting optimal and safe parameters requires careful consideration of the mechanisms through which cryotherapy affects swelling.

Postoperative swelling following TKA is primarily driven by inflammatory responses and haematoma, and cryotherapy works primarily on these mechanisms. All surgeries cause tissue damage, increase vascular permeability, and result in fluid exudation. However, in TKA, the bone cutting involved triggers a more intense inflammatory response, which is further exacerbated by ischaemia‐reperfusion injury caused by the use of a tourniquet during operation [23]. In addition to the usual bleeding caused by surgery, there are several other factors that can exacerbate haematoma formation. Tourniquets reduce visible bleeding during surgery but hinder identification of bleeding points, increasing postoperative bleeding risks. Muscle dissection adds to the bleeding, and anticoagulants for DVT prevention prolong bleeding time [26]. Postoperative rehabilitation activities further stress unhealed tissues, contributing to early swelling after TKA.

To establish standardised, effective, and safe cryotherapy parameters, this scoping review aims to analyze the available evidence on cryotherapy's role in controlling postoperative swelling after TKA, including the potential mechanisms of cryotherapy, its effects, and the evidence supporting the various treatment parameters, to help identify potential directions for future research.

## MATERIALS AND METHODS

### Scoping review: Identification of studies

A comprehensive literature search was conducted in PubMed, covering articles published from the inception of the database until 30 May 2024. The search strategy employed a combination of keywords and MeSH terms, with keywords being searched across “All fields”. The search string was as follows: (“cryotherapy” OR “cold therapy” OR “cryogenic therapy” OR “Cryotherapy” [MeSH Terms]) AND “knee” AND (“arthroplasty” OR “replacement” OR “TKA” OR “arthroplasty, replacement, knee” [MeSH Terms]) AND (“swelling” OR “edema” OR “inflammation”). Studies investigating cryotherapy in patients undergoing TKA were included, provided that swelling was one of the outcome measures, and the study described specific cryotherapy parameters. To ensure a comprehensive literature inclusion, any systematic reviews obtained from the search were examined for their referenced studies. If these studies were not retrieved in our initial search, they were considered additional resources for this review. Additionally, a snowballing approach was used to collect studies focused on cryotherapy parameters, even if they were not conducted in TKA patients, to help explain the cryotherapy parameter selection rationale.

## RESULTS

**Figure 1:**
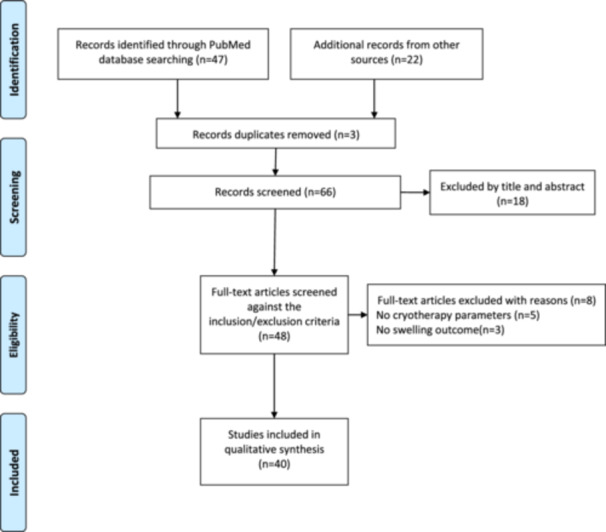
PRISMA flowchart.

A total of 69 studies were identified from the initial research, with 47 records from PubMed research and 22 records from other resources. Following a thorough screening, 40 articles were finally included for the full text analysis of the scoping review. The PRISMA flowchart for the scoping review is shown in Figure 1.

### Mechanisms of cryotherapy for early postoperative swelling after TKA

Cryotherapy can induce multiple physiological effects, including vasoconstriction, reduced capillary permeability, and decreased cellular metabolism. These effects lead to slowed blood flow, increased blood viscosity, reduced lymphatic flow, diminished connective tissue extensibility, and decreased nerve conduction velocity [25]. However, not all of cryotherapy's known effects are directly involved in reducing swelling after TKA. The key mechanisms at play in this context are as follows.

(1) Vasoconstriction and reduced haemorrhage: The most direct effect of cryotherapy is local vasoconstriction and muscle contraction, which decreases blood flow and perfusion, thereby reducing blood leakage. Although cryotherapy does not directly compress the blood vessels within the medullary cavity, it can still diminish blood extravasation by constricting the supplying blood vessels.
(2) Altered cell permeability and reduced tissue fluid leakage: By lowering tissue temperature and causing vasoconstriction, cryotherapy reduces the permeability of endothelial cells, leading to decreased leakage of plasma proteins, leucocytes and tissue fluid [8].
(3) Slowed activity of inflammatory cells and mediators: Cryotherapy lowers local temperatures and slows molecular movement, inhibiting the activity of inflammatory cells and the release of inflammatory mediators, thus mitigating the spread of inflammation [8].
(4) Metabolic slowdown: Post‐surgery, local tissues often experience stress due to insufficient blood supply and increased metabolic demand. Cryotherapy can help reduce this stress by lowering metabolism, which in turn decreases the production of reactive oxygen species and mitigates oxidative stress, thus alleviating tissue damage.
(5) Analgesic effects to reduce stress: The analgesic properties of cryotherapy help diminish the stress response caused by pain, indirectly lowering the intensity of the inflammatory response [24].

From this mechanistic analysis, it is evident that cryotherapy can primarily reduce swelling caused by haematoma or inflammatory responses, but it has little effect on swelling that has already formed. Moreover, this analysis allows researchers to evaluate the effectiveness of cryotherapy on swelling from a deeper perspective, focusing not only on measuring changes in lower limb circumferences post‐treatment but also analysing laboratory results or molecular dynamics, such as haemoglobin levels [1], inflammatory markers, blood flow perfusion levels and cellular activity rates.

### Effectiveness of cryotherapy in postoperative management following TKA

A systematic review from Cochrane Library [1], which included 22 trials up to 2022, examined the effectiveness of cryotherapy following TKA. The review, based on low‐quality evidence, suggests that cryotherapy may reduce postoperative blood loss by an average of 264 mL and slightly improve pain levels within the first 48 h post‐surgery, with a mean pain reduction of 1.6 points. Furthermore, it was found to enhance the range of motion at discharge, with an average increase of 8.3 degrees. Regarding swelling, evidence from seven studies (*n* = 403) demonstrated significant improvement in mid‐patellar swelling reduction between postoperative Days 2 and 6 (*p* = 0.001, *Z* = 3.21), with an average decrease of 7.32 mm (95% CI: −11.79 to −2.84). However, no improvements were observed at 6 weeks or 3 months postoperatively. Subgroup analysis revealed that the differences were primarily driven by two studies comparing combined cryotherapy and compression with a control group (*p* < 0.01, *Z* = 8.70). No significant differences were identified when comparing cryotherapy alone with compression (*p* = 0.41) or combined cryotherapy and compression with compression alone (*p* = 0.05). Thus, the evidence supporting the use of compression in reducing early postoperative swelling remains weak, with considerable heterogeneity in the parameters of cryotherapy and compression used across the included studies.

A meta‐analysis by Yu et al. [35] incorporated 10 studies from both Chinese and English literature and concluded that cryotherapy could reduce pain and haemoglobin decline within the first 2 weeks post‐TKA. However, it demonstrated no significant impact on reducing opioid consumption or improving joint range of motion. This suggests that cryotherapy may alleviate swelling by reducing haemoglobin loss and pain, consequently mitigating the formation of joint haematomas and inflammatory responses. In a systematic review by Bin et al. [4], no significant advantages were observed when comparing continuous compression cryotherapy systems to ice packs in reducing swelling at 24 h and 3 days post‐TKA. Nonetheless, by Day 7 postoperatively, continuous compression cryotherapy systems showed a more marked effect in swelling reduction. The divergence between this observation and theoretical expectations could be attributed to the lack of standardised, unified methods for assessing swelling, which poses challenges for data synthesis.

Additionally, a separate systematic review reported no significant difference in clinical outcomes between continuous cryotherapy devices and traditional cold therapy tools (such as ice packs or gel packs) in TKA patients. However, it is theoretically posited that continuous cryotherapy should improve outcomes by reducing inflammation, pain and swelling, thus facilitating faster recovery [20]. The inconsistent clinical outcomes may be attributed to various factors, including the level of cold penetration into tissues, cryotherapy methods, timing and duration of application, indicating that current cryotherapy protocols may not yet optimise therapeutic efficacy.

In summary, although evidence supports the effectiveness of cryotherapy in reducing early postoperative swelling following TKA, there remains no consensus regarding optimal parameters such as timing, temperature, frequency, and duration [4, 20, 35]. Further investigation is warranted to establish effective cryotherapy protocols and enhance its therapeutic benefits in the postoperative management of TKA.

## EFFICACY AND SAFETY OF CRYOTHERAPY PARAMETERS

### Timing of cryotherapy initiation

Current evidence indicates a trend towards earlier initiation of cryotherapy, with many studies now employing cryotherapy immediately following surgery [32]. Expert consensus further recommends administering cryotherapy for 10–15 min after each rehabilitation session [32]. Previous research has demonstrated that swelling increases by 35% on the first day after surgery [22], highlighting the necessity of early postoperative cryotherapy. Additionally, the first 2 h following TKA represent a critical period of rapid and severe ischaemia‐reperfusion [36], during which 37% of total blood loss occurs [15]. Based on the mechanism of postoperative swelling, inflammatory responses and bleeding begin immediately after surgery. Upon releasing the tourniquet, rapid vascular filling exacerbates bleeding, and haematoma formation further intensifies the inflammatory response. Therefore, initiating cryotherapy immediately after surgery may be crucial to mitigating these effects. However, in clinical practice, several challenges remain regarding the practical implementation of immediate postoperative cryotherapy. Specifically, questions persist about how to coordinate cryotherapy with wound dressing application, and how different dressing techniques may affect the efficacy of cryotherapy. These aspects warrant further exploration to optimise cryotherapy protocols in the postoperative setting.

### Temperature and frequency

The temperature and frequency of cryotherapy are critical factors influencing its therapeutic effectiveness. Some studies report the “treatment temperature” (i.e., the temperature of the cryotherapy device), while few provide detailed measurements of “skin temperature” or “intra‐articular temperature”. When cryotherapy is applied at the treatment temperature, it lowers the skin temperature, which in turn decreases intra‐articular temperature. Once the intra‐articular temperature reaches an “effective” range, cryotherapy begins to exert its effects, reducing blood flow and inflammatory activity. However, if the intra‐articular temperature continues to drop beyond a “dangerous” threshold, it may cause vascular or skin damage. Therefore, the duration of each cryotherapy session must be limited to prevent temperatures from reaching harmful levels. The interval between sessions, or the recovery time, refers to the period after a cryotherapy session during which the skin and intra‐articular temperatures gradually return to levels above the “effective” range. Once the temperature has sufficiently recovered, the next round of cryotherapy can be initiated to maintain the therapeutic effects. Thus, defining the “effective” and “dangerous” thresholds for treatment temperature, skin temperature, and intra‐articular temperature is crucial for determining optimal cryotherapy parameters.

#### Intra‐articular temperature

**Figure 2:**
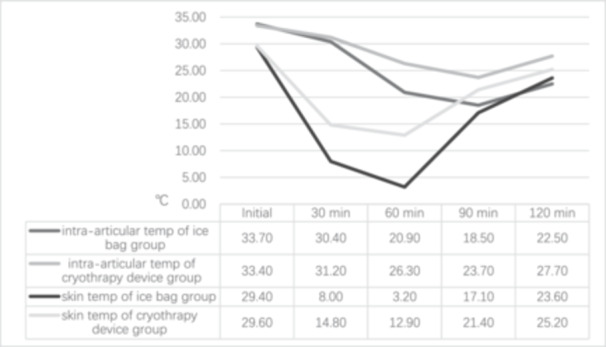
Effects of two cryotherapy devices on skin and intra‐articular temperatures of the knee [31].

Due to the risk of infection from using temperature probes to measure intra‐articular temperature following TKA, which could lead to implant failure, there are no studies specifically exploring intra‐articular temperature during cryotherapy after TKA. Only a few studies have investigated intra‐articular temperatures in healthy individuals or in patients undergoing other knee surgeries. Glenn et al. [11] measured the changes in suprapatellar pouch, lateral gutter, and skin temperatures in patients undergoing anterior cruciate ligament reconstruction, comparing a cryotherapy group to a non‐intervention group. The initial temperatures for both skin and intra‐articular regions were approximately 27°C. In the group without cryotherapy, skin temperature increased by 0.01°C, while the temperatures in the suprapatellar pouch and lateral gutter increased by 3.3°C and 3.4°C, respectively. In contrast, after 1 h of cryotherapy, skin temperature decreased by 12.3°C, the suprapatellar pouch temperature dropped by 2.7°C, and the lateral gutter temperature remained unchanged. However, cryotherapy lowered the suprapatellar pouch temperature by 6.0°C, and although the lateral gutter temperature did not decrease further, cryotherapy prevented the natural rise of 3.4°C observed without intervention. Another study explored the impact of different cryotherapy devices on the skin and intra‐articular (suprapatellar pouch) temperatures in healthy individuals [31]. The temperature trends reported in this study are illustrated in the following graph (Figure 2). From these data, the following conclusions can be drawn: (1) During both cooling and rewarming phases, the rate of skin temperature change is rapid at first but slows over time, while intra‐articular temperature changes most rapidly between 30 and 60 min, with slower changes before and after this period; (2) the rate of intra‐articular temperature change (4.4°C/h–19°C/h) is significantly lower than that of skin temperature (3.8°C/h–42.8°C/h) and (3) intra‐articular temperature changes occur with a delay. Skin temperature reaches its lowest point at 60 min post‐cryotherapy (3.2°C) and then gradually rises as the effect of cryotherapy diminishes (e.g., melting of the ice pack). In contrast, intra‐articular temperature reaches its lowest point at 90 min post‐cryotherapy (18.5°C). Although some studies have reported trends in intra‐articular and skin temperature changes, the precise relationship between the two remains unclear. Moreover, no consensus exists regarding the optimal effective intra‐articular temperature for cryotherapy.

#### Skin temperature

Due to the challenges in measuring intra‐articular temperature and the lack of strong evidence for an effective intra‐articular temperature range, many researchers have instead focused on exploring effective skin temperatures to define the efficacy of cryotherapy. There is theoretical evidence suggesting that a skin surface temperature of 13.5°C or lower confirms pain relief following cryotherapy, but there is currently insufficient evidence to identify a target temperature for reducing swelling [9]. Belsey et al. [2] proposed that intra‐articular temperature must drop below 30°C to reduce post‐traumatic inflammatory metabolic activity, and that significant intra‐articular changes begin when skin temperature falls below 20°C. They further identified a skin temperature range of 10°C–15°C as optimal for cryotherapy to exert its effects, with minimal risk of frostbite or other injuries. Based on this evidence, it may be reasonable to define an effective skin temperature for cryotherapy as 10°C–15°C, which can serve as a target for establishing other cryotherapy parameters. Skin temperature is influenced by various factors, including treatment temperature, duration of application, coverage area, and the degree of compression applied. All of these factors should be considered when determining optimal treatment settings.

#### Therapeutic temperature

Some researchers report only the cooling modality used, such as ice‐water mixtures, without specifying the exact therapeutic temperature [6, 34, 37]. Studies utilising computer‐assisted cryotherapy devices tend to specify the therapeutic temperature. In Yanfei et al.'s [32] evidence summary, there is one piece of evidence for a therapeutic temperature of 6°C, and two pieces each for 8°C, 10°C and 12°C, indicating a lack of consensus. A study comparing the effects of five different pressurised cryotherapy devices on skin temperature found that only two devices reduced skin temperature to below 15°C after 20 min of therapy. These devices were set at a therapeutic temperature of 8°C with 15–50 mmHg of pressure, and 1°C with 5–50 mmHg of pressure, respectively. However, for the other three devices, the skin temperature remained above 15°C even after 30 min of treatment, failing to reach the target temperature [3]. This evidence provides useful reference points for the combination of therapeutic temperature, pressure and duration settings. Another study using computer‐assisted cryotherapy devices with different therapeutic temperatures (6°C, 8°C, 10°C and 12°C) reported that the difference between skin temperature and the therapeutic temperature after 30 min of treatment ranged from 5.7°C to 6.8°C. This suggests that a stable temperature gradient can be used to set the therapeutic temperature [2]. However, a study evaluating the performance of 13 different cryotherapy devices found significant variation in surface temperature distribution across different areas of the devices. The authors argued that representing therapeutic temperature with a single value is inadequate [14]. The discrepancies in achieving therapeutic temperature across different cryotherapy devices may be attributed to the varying methods of cooling transfer. These methods include convection, conduction, and evaporation. For example, cold packs/ice bags, ice massage and cold towels primarily use conduction, where heat exchange occurs through direct contact between two substances at different temperatures. Cold baths involve convection but are not suitable for TKA patients. Cryospray utilises evaporation, where heat is absorbed from the body during the vaporisation process. Lee et al. [17] used cryotherapy by spraying liquid carbon dioxide at −78°C from a distance of approximately 10 cm from the knee joint. Thus, when determining therapeutic temperature, the cooling transfer method and the difference between the set and actual temperature should also be considered.

#### Duration of each session

In the evidence summary by Yanfei et al. [32], the duration of cryotherapy sessions in the 17 included studies varied from the traditional 20 or 30 min. Instead, there were two pieces of evidence each for durations of 2, 4, and 6 h, and one piece each for 12‐h and 24‐h sessions. This variation may be attributed to the differences in the ability of various cryotherapy devices to maintain low temperatures over time, with computer‐assisted devices typically able to sustain longer application periods. Some researchers have designed stepwise cryotherapy protocols, as shown in Table 1. These protocols generally follow the principle of starting with a lower temperature and longer duration, and then gradually increasing the temperature while shortening the session duration. This approach aims to better control early postoperative swelling, although a standardised protocol has yet to be established.

**Table 1:** Stepwise cryotherapy protocol parameters following TKA.

Literature	Time point	Duration per session (h)	Temp (°C)	Freq. (times/day)
Brouwers et al. [7]	IPO	6	6	1
	POD 1	4	8	3
	POD 2‐7	2	8–10	3
Thijs et al. [30]	IPO	6	10	2
	POD 1‐7	2, 4 h at night	10, 12°C at night	3
Sadoghi et al. [27]	Pre‐op Day 1	≥1	‐	1
	IPO	6, 4 h at night	‐	2
	POD 1‐6	2, ≤4 at night	‐	3

Abbreviations: IPO, immediate postoperative period; POD, postoperative day; Pre‐op, preoperative day; TKA, total knee arthroplasty.

Some studies have provided evidence supporting the need for extended cryotherapy durations. Since skin temperature takes 20 min or more to reach the effective range after the start of cryotherapy, the duration of the treatment does not necessarily equate to the time during which the patient benefits from it [3]. However, other researchers argue for shortening cryotherapy sessions. Khoshnevis et al. [13] found that even after cryotherapy ends and skin temperature returns to baseline, blood perfusion remains low, which could potentially lead to tissue damage. By quantifying the extent and duration of vasoconstriction after cryotherapy, they observed that following 60 min of 3°C–5°C cryotherapy, skin temperature slightly decreased below 10°C, and the treated area reached a state of deep vascular constriction. During the 90‐min rewarming period after cryotherapy cessation, the local temperature rose to 20°C–22°C, but blood perfusion showed no significant increase, nor was there cumulative enhancement. This suggests that the maintenance of vasoconstriction is not directly dependent on the continued presence of cold; rather, the extent and prolongation of vasoconstriction may induce an ischaemic state equivalent to non‐freezing cold injury. These findings highlight the need for careful consideration when determining the optimal duration of cryotherapy sessions to ensure both efficacy and safety.

From an efficacy standpoint, some studies have shown that extended cryotherapy using computer‐assisted devices produces better outcomes in early postoperative joint mobility compared to traditional cryotherapy (e.g., 30‐min ice pack applications, three times per day) [27]. A meta‐analysis revealed that continuous cryotherapy is more effective than intermittent cryotherapy in reducing peak pain at 48 h, improving joint mobility, reducing blood loss and decreasing the need for additional analgesics. However, during continuous cryotherapy, it is crucial to monitor skin temperature, with 10°C being the critical threshold [28]. From a safety perspective, Thijs et al. [30]. reported that 10% of patients in the low‐temperature group (*n* = 3) experienced discomfort during overnight 4‐h sessions at 10°C–12°C, with some reporting generalised cold sensations. Thus, the establishment of standardised, effective, and safe durations for cryotherapy sessions remains to be determined [18].

#### Intermittent duration

The cooling temperatures achieved and the duration of cryotherapy both influence the rate of blood perfusion changes during the rewarming phase. The purpose of intermittent cryotherapy is to allow partial recovery of blood perfusion levels, thereby avoiding potential damage from prolonged cooling and reduced perfusion. However, previous studies have shown that changes in skin temperature during the rewarming phase do not directly correlate with perfusion recovery. For instance, after cryotherapy, even when skin temperature rises back to 20°C–22°C during rewarming, no significant change in blood perfusion was observed when the skin temperature had previously dropped below 10°C, and no cumulative increase in perfusion occurred [13]. Therefore, skin temperature alone cannot reliably represent blood perfusion levels. This finding suggests that other indicators should be explored, or foundational research could be conducted to establish a model that correlates low skin temperature and its duration during the rewarming phase with the optimal duration of intermittent periods. Such a model could provide valuable guidance for clinical practice.

### Coverage area

Some studies do not specifically report the coverage area of cryotherapy applications. Traditional ice packs are usually limited in size. To address this limitation, Yingchao et al. [34] made a cryotherapy wrap that increased the surface area covered by a single ice pack, while also applying compression, resulting in improved therapeutic outcomes. Computer‐assisted cryotherapy systems and pressurised ice packs generally offer a larger coverage area, often using a wrap‐around design that enhances comfort and convenience [24]. Although studies have explored the effects of different cryotherapy devices [20], comparisons based on coverage area have not been reported. Theoretically, a larger cryotherapy area would accelerate the achievement of target temperatures. Therefore, while ensuring cryotherapy safety, the primary consideration could be the cost of devices that cover the target treatment area.

### Site

When selecting the application site for cryotherapy, the primary considerations are therapeutic efficacy and safety, followed by comfort and convenience. The standard incision for TKA is a midline incision, so most studies apply cryotherapy to the anterior knee. A few studies have explored applying cryotherapy to the posterior knee, which, although seemingly more convenient, may increase discomfort [18]. In terms of effectiveness, Foster et al. [10] reported that after placing ice around the wound following knee replacement surgery, skin blood flow decreased by approximately 40%. Ice placement over the incision reduced blood flow more significantly than placement behind the knee (*p* < 0.05). Bingdu et al. [5] have suggested placing ice packs on the knee while avoiding the patella to prevent disrupting blood flow around the wound, and steering clear of the popliteal fossa to avoid pressure on the major blood vessels and nerves behind the knee. This choice of application site is worth considering.

### Duration of cryotherapy

The duration of cryotherapy varies across studies, ranging from 2 days to 1 week or until discharge [18]. According to a meta‐analysis, cryotherapy can improve pain levels up to 2 weeks postoperatively, leading some researchers to recommend extending cryotherapy for 2 weeks after surgery [35]. Determining the optimal duration of cryotherapy is crucial for maximising patient outcomes while controlling healthcare costs and workload. Analysing the mechanism of cryotherapy's effect on swelling, its primary impact is reducing bleeding and controlling the inflammatory response. The appropriate duration can be identified by examining post‐TKA changes in haemoglobin levels and inflammatory markers, such as CRP. A systematic review of inflammatory markers after TKA revealed that significant changes in CRP and IL‐6 levels reflect inflammation and clinical outcomes. CRP typically peaks 1–3 days after surgery and returns to baseline within 2 weeks to 1 month [21]. This pattern suggests that controlling inflammation with cryotherapy is necessary before the CRP peak, and even when CRP starts to decline but remains elevated, continued cryotherapy may still be needed to manage inflammation, though the frequency could be reduced until the inflammation subsides. While it remains to be studied whether cryotherapy can shorten the time it takes for CRP to return to baseline, theoretically, cryotherapy could reduce CRP peak levels. Research on postoperative blood loss following TKA often focuses on changes in haemoglobin levels during the first 1‐5 days after surgery [12]. Current guidelines recommend continuing anticoagulant use for at least 1 week postoperatively, so cryotherapy for haematoma prevention could align with the duration of anticoagulation therapy [26]. Thus, theoretical analysis suggests that the first week post‐surgery may be a critical period for cryotherapy, after which the frequency or duration can be reduced. By 1 month postoperatively, the risk of inflammation and haematoma formation is significantly lower, and cryotherapy could be discontinued. Based on this analysis, future studies could design and validate cryotherapy protocols.

### Limitations

It is acknowledged that this review has certain limitations. Firstly, despite efforts made to search and screen relevant literature, some studies may have been omitted due to search strategies, database constraints, or specified timeframes. To reduce this discrepancy, a snowballing retrieval approach was adopted, aiming to maximise the acquisition of valuable literature and evidence. Additionally, the inclusion of literature without a quality assessment may have affected the level of evidence. Lastly, while some insights have been gained, unresolved issues remain, which may pose obstacles for clinicians attempting to implement cryotherapy interventions. Therefore, it is suggested that future research endeavours to fill these gaps.

## CONCLUSION

Cryotherapy helps control postoperative swelling after TKA primarily by reducing bleeding and inflammatory responses. Mechanism analysis assists in identifying objective indicators for evaluating cryotherapy's effectiveness. Although low‐quality evidence suggests cryotherapy is effective for early postoperative swelling, significant heterogeneity exists in the parameters used. Understanding the characteristics of swelling causes, such as the pattern of inflammatory marker changes and anticoagulant duration, may help define some parameters (e.g., timing and duration). While measuring intra‐articular temperature is challenging, evidence supports the efficacy of cryotherapy at reducing skin temperature. However, determining the optimal combination of treatment temperature, pressure and duration to achieve target skin temperature remains unclear. Once target skin temperature is established, further exploration of blood perfusion levels during rewarming is needed to clarify cryotherapy's single application duration and intermittent timing. Ultimately, selecting feasible, efficient, and cost‐effective cryotherapy devices based on these parameters will improve therapeutic outcomes and reduce the peak, rate, and incidence of early postoperative swelling after TKA.

## AUTHOR CONTRIBUTIONS

**Lin Yang**: Literature search; scoping review; writing and editing the article. **Yi‐fang Zhan**: scoping review; writing and editing the article. **Zan‐jing Zhai**: editing the article. **Hong Ruan**: editing the article. **Hui‐Wu Li**: editing the article.

## CONFLICT OF INTEREST STATEMENT

The authors declare no conflicts of interest.

## ETHICS STATEMENT

Not required as this is a scoping review.

## Data Availability

The data from the present study is available and can be shared upon reasonable request.
